# Modeling cholinergic retinal waves: starburst amacrine cells shape wave generation, propagation, and direction bias

**DOI:** 10.1038/s41598-023-29572-2

**Published:** 2023-02-17

**Authors:** Matthew J. Tarchick, Dustin A. Clute, Jordan M. Renna

**Affiliations:** Department of Biology, University of Akron, Akron, OH 44325-3908 USA

**Keywords:** Retina, Neuronal development

## Abstract

Stage II cholinergic retinal waves are one of the first instances of neural activity in the visual system as they are present at a developmental timepoint in which light-evoked activity remains largely undetectable. These waves of spontaneous neural activity sweeping across the developing retina are generated by starburst amacrine cells, depolarize retinal ganglion cells, and drive the refinement of retinofugal projections to numerous visual centers in the brain. Building from several well-established models, we assemble a spatial computational model of starburst amacrine cell-mediated wave generation and wave propagation that includes three significant advancements. First, we model the intrinsic spontaneous bursting of the starburst amacrine cells, including the slow afterhyperpolarization, which shapes the stochastic process of wave generation. Second, we establish a mechanism of wave propagation using reciprocal acetylcholine release, synchronizing the bursting activity of neighboring starburst amacrine cells. Third, we model the additional starburst amacrine cell release of GABA, changing the spatial propagation of retinal waves and in certain instances, the directional bias of the retinal wave front. In total, these advancements comprise a now more comprehensive model of wave generation, propagation, and direction bias.

## Introduction

Theoretical models and experimental examination have had a profound effect on the study of neurons and will continue to direct efforts for examining complicated aspects of neural transmission. In 1952, Hodgkin and Huxley formulated electrical equivalence of the giant squid axon^1^ creating a model that could explain the sources of ionic currents in neurons. Since the introduction of electrical equivalence, theoretical neuron models have become useful for demonstrating the foundations of neural concepts that allude to experimental setups. A simplification to the Hodgkin Huxley model called the Morris-Lecar model allowed for a better understanding of time delayed voltage oscillations. This model also introduced bifurcation analysis to the original equations^2,3^. As models became more descriptive, experiments could also increase their scope. One such example of this relationship between experimentation and theory is stage II cholinergic retinal waves. These waves exist during a developmental timepoint in which network properties of the retina are still taking shape. They are nearly ubiquitous in mammals and act to establish the visual circuits^4–6^. One of the earliest theoretical models of retinal waves accurately predicted the existence of a network of neurons in the retina that could be used for establishing topographic maps^7^.

Indeed, the establishment of efficient neural code in the retina and brain requires spontaneous activity for assembly and construction^8^. A key characteristic of retinal waves is that their presence precedes light evoked outer-retinal responses^9^. By the time visual stimuli are present, many of the neuronal networks are well established and need only fine tuning^10^. After the phenomenon was observed in a living animal^11^, incremental steps between theory and experiment have generated more concise models. In 1995, calcium imaging indicated that spontaneous activity in the retina occurs in synchronized calcium “bursts” in the ganglion cells^12^. Similar activity occurs in other layers of the retina. These calcium bursts are part of a larger wavelike behavior that propagate across the retina^13^. These waves were found to coincide with the extracellular release of acetylcholine^14,15^, rarely overlapping and occurring all throughout retina^16^. Early computational models utilized these findings to build more descriptive models of propagation and refractoriness^17^. For example, the population of cells responsible for initiation and propagation of retinal waves was determined to be a network of cells presynaptic to ganglion cells called the starburst amacrine cells (SAC)^15^. When modelling these cells as a 2D lattice, their combined network dynamics were found to contribute to wave-like propagation and termination without the need for inhibition^17^.

Additional physiological mechanisms of the SACs were uncovered. Cells within this network contain a high degree of stochastic "noisy" activity, and each SAC during the early stages of development is capable of spontaneously firing without a stimulus. SAC firing is characterized by short “spikelets” sitting atop a 1–2 s "burst" of calcium activity, followed by 15–30 s of hyperpolarization known as the slow after-hyperpolarization (sAHP)^18^. Incidentally, the length of the sAHP is directly proportional to the amount of time the cell spends in a depolarized state^16^. The synchronized firing of SACs is the result of the release of acetylcholine. Previous models utilize a Crank–Nicholson diffusion scheme, which is released diffusely into the extracellular space between SAC and retinal ganglion cell (RGC) dendritic fields^18,19^. Theoretical models describing the lateral diffusion of acetylcholine within the inner nuclear layer (INL) also supported the wavelike properties of cholinergic retinal waves^20^. It was also hypothesized that during the propagation of retinal waves, a certain percentage of SACs do not synchronize with the other cells, although the exact mechanism for this phenomenon remains unknown^18^.

The propagation of stage 2 retinal waves is driven by acetylcholine release, but the role of GABA is not known. SACs are unique in their ability to secrete both acetylcholine and GABA^15,21,22^. Initially GABA does not act as a hyperpolarizing neurotransmitter^23^. This was found to occur as early as embryonic day 30 (E30) in the rabbit retina^22^. The application of GABA agonists at early stages in development results in calcium efflux in the SAC cell layer indicating depolarization^22^. This is because of the expression of potassium-chloride cotransporters. In rats for example these cotransporters become expressed after the second postnatal week^24^. Removing the influence of these channels by blockade results in a higher excitability of the SAC. Removing chloride from the extracellular solution also results in a similar phenomena^23^. The equilibrium potential of chloride has an important developmental function. Including GABA and the chloride equilibrium potentials into a computational model could improve understanding of how the SAC layer can generate and propagate retinal waves.

GABA is a critical neurotransmitter driving directional selectivity in the mature retina. How this was established is not entirely known. GABA release may not be confined to a specific subregion of the SAC, but it may be asymmetrically released^25^. Muscimol, and activator of GABA receptors, blocks spontaneously initiated retinal waves^26^. The addition of GABAzine further removes directionality of retinal waves and removing two varieties of directionally sensitive ganglion cells downstream from SACs does not alter the directionality^27^. Removal of b2 nicotinic acetylcholine receptors also disrupts the directionality, but the spatiotemporal properties are not altered^28^ .Previous models describe the release of acetylcholine^20^, but to our knowledge no model has been proposed to explain the biophysical properties of the co-release of both acetylcholine and GABA.

Similarly, the underlying mechanism for the sAHP has also not fully been described. Techniques such as mRNA sequencing and electrophysiology suggest that two-pore potassium channel called two-pore domain weak inwardly rectifying K channel (TWIK)-related potassium channel-1 (TREK1) could be a potential generator of the sAHP^29^. TREK1 is highly expressed in SACs at postnatal day 6 (P6), when retinal waves are reaching peak activity. Activation of adenylyl cyclase, as well as the increased production of cAMP, influences these channels by altering the frequency of retinal waves. Moreover, retinal waves were unaltered by addition of apamin, an agonist of the small conductance calcium dependent potassium channel SK1^29^ to which previous models have misattributed the sAHP. TREK1 is inactivated by the process of phosphorylation of two serine sites S-300 and S-333 on the intracellular portion of TREK1, facilitated by protein kinase A (PKA)^30^. PKA and cAMP oscillate inversely to calcium influx in dissociated cell cultures in RGCs as well as SACs^31^. As such, the decrease in cAMP, followed by gradual dephosphorylation (and thus activation) of TREK1 is a possible mechanism for the generation of the sAHP. A more extensive reaction network could better connect the relationship between Calcium influx and TREK1 activation, and better define the relationship between calcium bursts and the sAHP length^32^.

This model extends and clarifies previous models in three ways. The first is to propose a model of slow afterhyperpolarization (sAHP) generation. The sAHP is generated by calcium dependent decay of cAMP which activates TREK1 potassium channels. By including biologically relevant targets in this model, the potential to expand the model to other signaling pathways becomes a future direction for modelling. The inclusion of slow second messenger processes utilizing cAMP and TREK1 variables will make this model more consistent with the intracellular biochemical reactions of a realistic cell. The second is to describe the dynamics of the SAC synchronization through a cholinergic network. By including the lateral diffusion of acetylcholine and cholinergic neighboring SACs synchronize and can pass on bursting activity while SACs further apart will be less likely to synchronize. The third is to describe a process by which GABAergic currents may asymmetrically inhibit waves and enforce directionality of retinal waves. This could potentially explain how directional retinal wave propagation occurs in the SAC layer.

## Results

### Spiking metrics of model components

**Figure 1:**
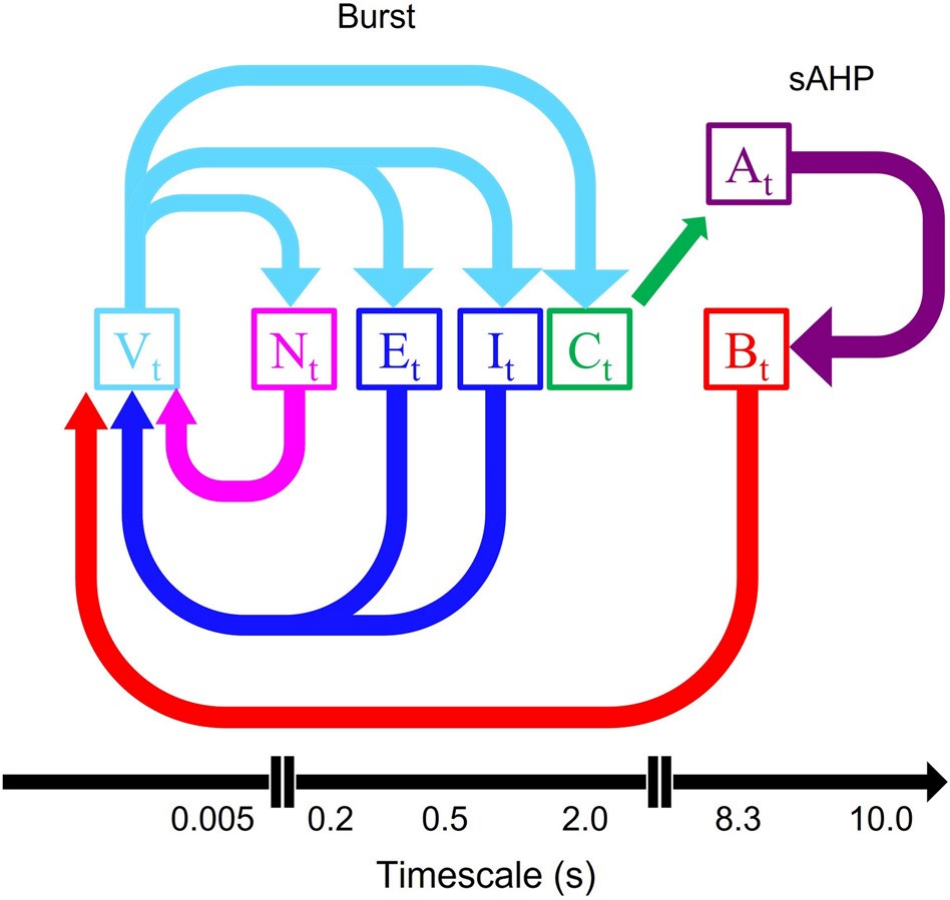
Model variables and relationships. A schematic of the relationships in the model. Each arrow represents the effect on a variable. Membrane voltage (*V*_*t*_) causes changed in potassium opening probability (*N*_*t*_), acetylcholine release (*E*_*t*_), GABA release (*I*_*t*_), and calcium influx (*C*_*t*_). Calcium affects cAMP concentration (*A*_*t*_) and TREK1 opening (*B*_*t*_). TREK1 (*B*_*t*_), acetylcholine (*E*_*t*_), GABA (*I*_*t*_), and potassium opening probability (*N*_*t*_), then affects voltage.

Each term in the model was grouped into three-time scales, given by their time constants (*τ*): fast (5*.*0 − 20*.*0 ms) medium (0*.*5 s*–*2 s) and slow (> 15 s) (Initial conditions for each term are outlined in Table 1). The model was run with fixed parameters (outlined in Table 2). This also will set the four metrics used to summarize the model: spikelet length and interval (fast), burst duration (medium) and burst interval (slow) (Fig. 1).

**Table 1:** Initial conditions and values used in the model.

Parameter	Initial condition	Unit
*V*_*t*_	− 63.6	mV
*N*_*t*_	0.0	
*M*_*t*_	0.062	
*H*_*t*_	0.550	
*C*_*t*_	0.085	mM
*A*_*t*_	0.026	
*B*_*t*_	0.0	
*E*_*t*_	0.066	mM
*I*_*t*_	0.053	mM
*W*_*t*_	0.0	pA

**Table 2:** Initial values and units of each of the biophysical parameters used in the model.

Parameter	Value	Unit
*V*_1_	− 20.0	mV
*V*_2_	20.0	mV
*V*_3_	− 25.0	mV
*V*_4_	7.0	mV
*V*_*0e*_	− 40.0	mV
*V*_*se*_	0.2	mV
*V*_*0i*_	− 40.0	mV
*V*_*si*_	0.2	mV
*V*_*7*_	10.0	mV
*V*_*8*_	− 40.0	mV
*V*_*9*_	10.0	mV
*V*_*10*_	4.0	mV
*V*_*11*_	− 65.0	mV
*V*_*12*_	18.0	mV
*V*_*13*_	0.07	mV
*V*_*14*_	− 65.0	mV
*V*_*15*_	20.0	mV
*V*_*16*_	1.0	mV
*V*_*17*_	− 35.0	mV
*V*_*18*_	10.0	mV
*C*_*m*_	17.0	pF
*g*_*Leak*_	2.0	nS
*g*_*Ca*_	8.5	nS
*g*_*K*_	4.0	nS
*g*_*Na*_	2.0	nS
*g*_*TREK*_	2.0	nS
*g*_*ACh*_	0.215	nS
*g*_*GABA*_	0.9	nS
*σ*	5.0	pA
*E*_*Leak*_	− 70.0	mV
*E*_*Ca*_	50.0	mV
*E*_*K*_	− 90.0	mV
*E*_*Na*_	55.0	mV
*E*_*ACh*_	0.0	mV
*E*_*Cl*_	− 65.0	mV
*τn*	5.0	ms
*τa*	8300.0	ms
*τb*	8300.0	ms
*τC*	2000.0	ms
*τACh*	540.0	ms
*τGABA*	1000.0	ms
*τW*	1000.0	ms
*C*_0_	0.088	mM
*λ*	2.702	mM
*δ*	0.010503	mM ∗ pA^−1^
*α*	625.0	–
*β*	34.0	–
*ρe*	6.0	mM ∗ pA^−1^
*ρi*	5.0	mM*pA^−1^
*K*_*d*_	0.1	mM^−2^
D_e_	0.005	mm ∗ s^−1^
D_i_	0.005	mm*s^−1^
*dXi {L,R}*	{1.0, 1.0}	
*dXe {L,R}*	{1.0, 1.0}	
*dYi {U,D}*	{1.9, 0.1}	
*dYe {U,D}*	{1.0, 1.0}

**Figure 2:**
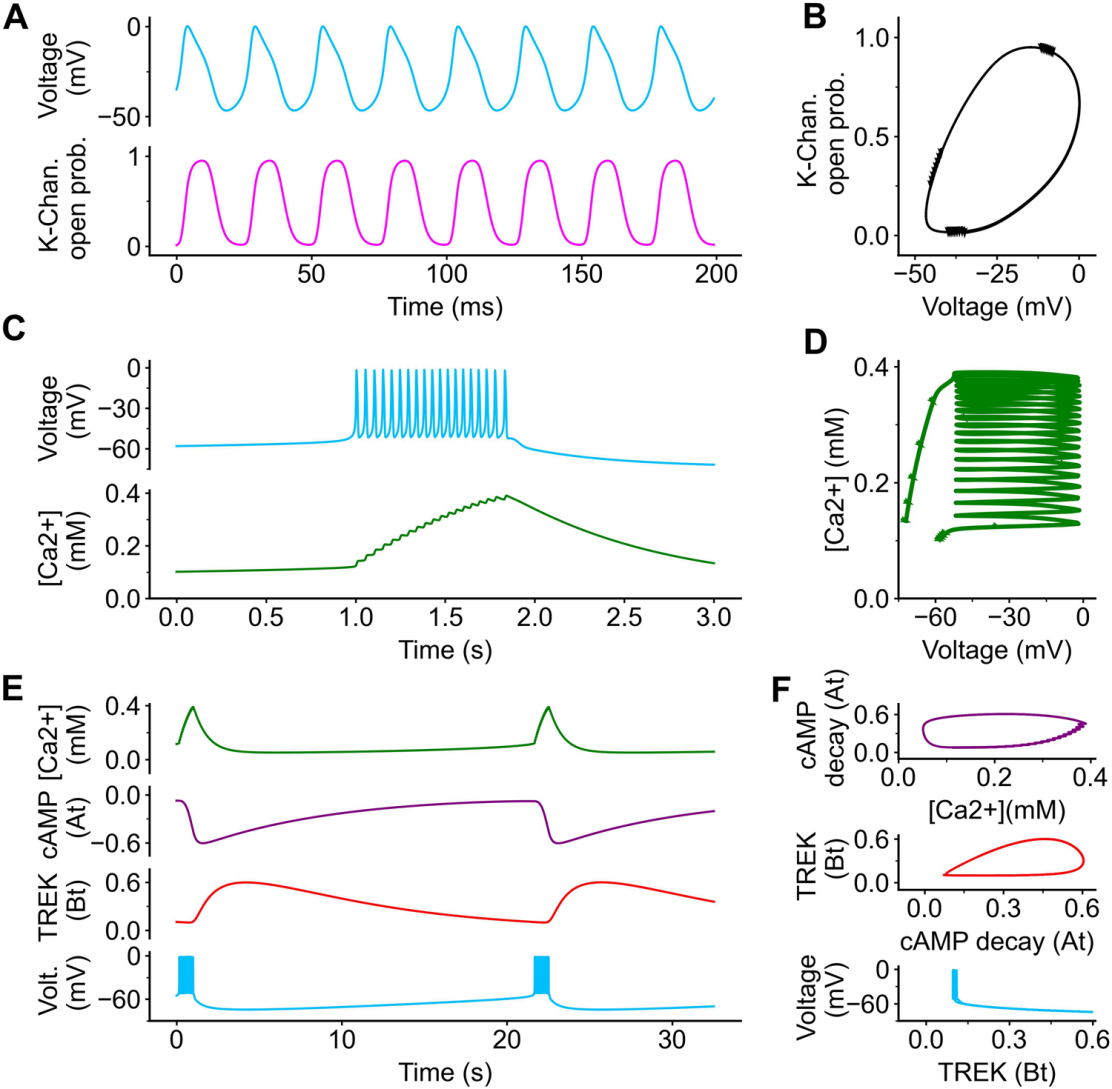
Model traces and oscillations. A 10pA current is added (the equivalence of current clamping a cell) to replicate spiking behavior. In (**A**) As the membrane voltage evolves (*V*_*t*_), the potassium channel opening ratio (*N*_*t*_). (**B**) These variables (V_*t*_ and *N*_*t*_) form a oscillating cycle. (**C**) As the voltage (*V*_*t*_) evolves, the influx of intracellular calcium begins to increase (*C*_*t*_). The time scale in (C) is in seconds. (**D**) These variables (V_*t*_ and *C*_*t*_). also oscillate, but the calcium concentration increases at the same time. (**E**) As calcium (*C*_*t*_) evolves, cAMP/cAMP decay (*A*_*t*_), and TREK1 channel activation (*B*_*t*_). (**F**) These variables oscillate and eventually lead to an afterhyperpolarization.

The fast spikelets (Fig. 2A) are driven by the oscillatory relationship between the membrane voltage (*V*_*t*_) and the proportion of open voltage gated potassium channels (*N*_*t*_). The first and central variable modelled is voltage (*V*_*t*_). The general outline for the voltage is an additive conductance equation that have been modeled previously^1,3,33^. The voltage equation utilizes channel conductance, but also uses an experimentally derived parameter to set the rate at which the cells can form spike. The variable *N*_*t*_ is a dimensionless ratio that describes the proportion of the voltage gated potassium channels open. A value of 1 means that the cell has a strong voltage gated potassium current and will very quickly return to a baseline. The speed at which spiking occurs is strongly influenced by the rate at which the potassium channels open (Λ, voltage dependent) and the decay rate at which 100% of the potassium channels close (*τ*_*N*_ = 5* ms*). Activation and decay set the spike duration and interval of the cell and is what we call "spikelets” in the analysis. The shape of the limit cycle (Fig. 2B) gives information about the properties of this spiking cycle. The narrower the shape of this limit cycle, the more quickly the cell’s voltage repolarizes and the shorter the spiking interval.

The spikelets can be seen on top of a slower rising event, or the "burst" (Fig. 2C). Calcium release (*C*_*t*_) is proportionally dependent on voltage. An important aspect of this model is the relationship between the time the cell spikes and the amount of calcium (Fig. 2D). This spiking continues to increase calcium load until the sAHP is triggered. This marks the end of the measured burst duration. The sAHP is mediated by 2 independently evolving variables, a variable representing the decay of a second messenger cAMP (*A*_*t*_) and a variable representing the activation of TREK1 channels (*B*_*t*_) (Fig. 2E). These variables are involved in secondary limit cycles (Fig. 2F). Once enough TREK1 channels open, a negative current overpowers the voltage completing the cycle. The interburst interval begins at the end of the burst and ends when a new burst has started.

### Dynamic analysis, spiking threshold, and the slow afterhyperpolarization

**Figure 3:**
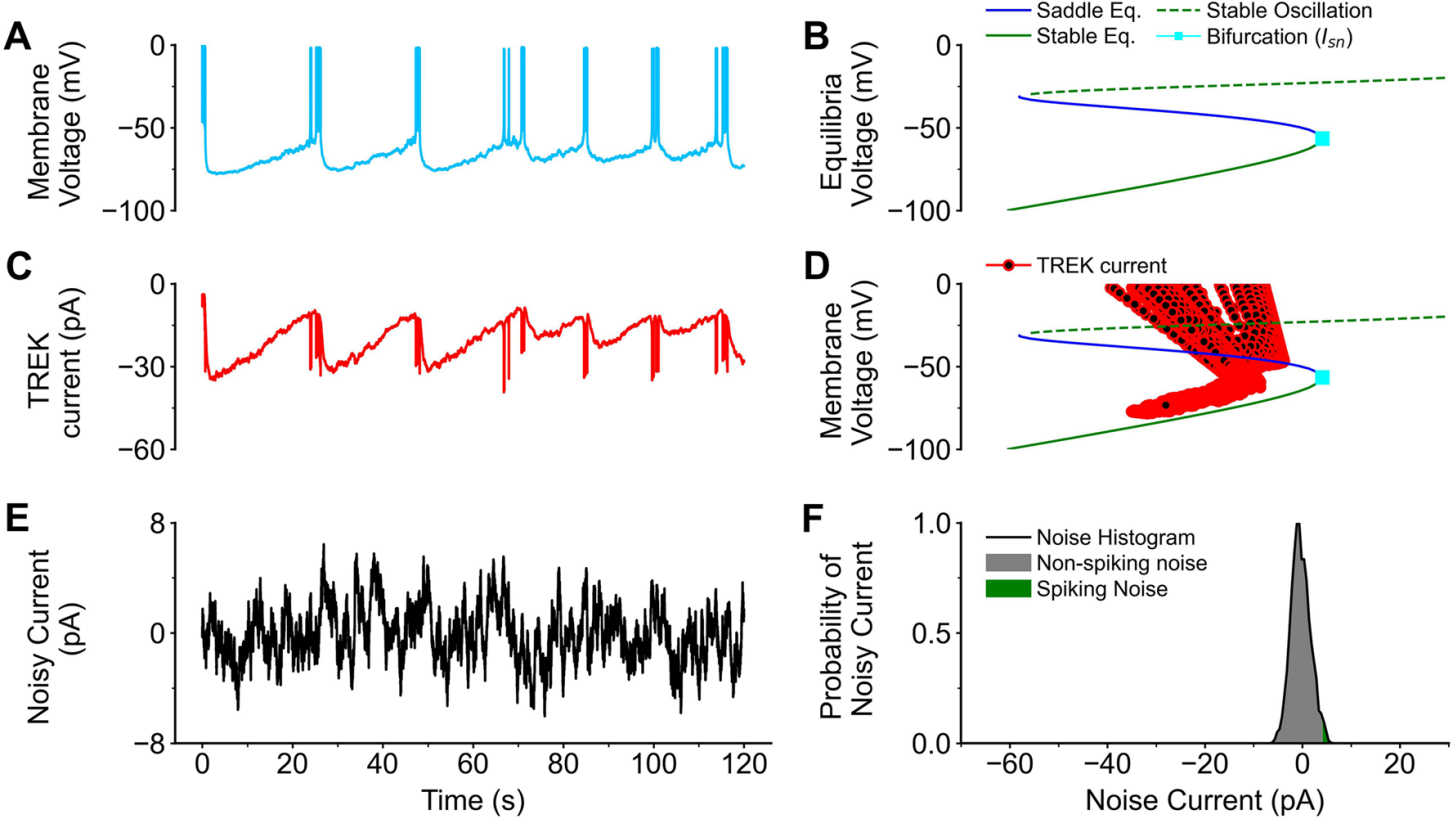
Biophysical properties of starburst amacrine cell. The biophysical properties of starburst amacrine cells (SAC) are driven by spontaneous noise and the slow-afterhyperpolarization (sAHP). (**A**) After noisy channel activity and TREK channels are added in, the membrane voltage (*V*_*t*_) forms spikelet and bursts separated by an interburst interval. (**B**) A codimensional-1 analysis was conducted altering the applied current (I_app_) without the influence of acetylcholine, GABA, or the sAHP (g_*ACh*_ = 0, g_GABA_ = 0, g_TREK_ = 0). Lines on the graph indicate the presence of equilibria. Green lines indicate a stable equilibrium. Dashed lines indicate that the equilibria will be the focus of a oscillation. Blue lines indicate a Saddle equilibrium exists. The location of the saddle node current (I_sn_ = 4.09, V_sn_ = ** − **56.6) represents the excitability threshold. (**B**) TREK1 channels interact with the voltage and are responsible for the sAHP. (**D**) The relationship between the voltage and the sAHP (red line) are overlayed on the equilibria plot. (**C**) Noisy channel activity is added to the model as a current in pA. (F) A histogram samples the distribution of noisy currents. The green shaded area represents spikes that are above the I_sn_ and capable of generating a spike.

After the addition of noisy channel activity, the cell is capable of spontaneous bursts (Fig. 3A). During these bursts the variables *V*_*t*_ and *N*_*t*_ form an oscillating limit cycle Fig. 2A). Previous models have used bifurcation analysis to validate the presence of spiking vs non-spiking activity in the form of a limit cycle or oscillation^34^. This limit cycle can be predicted by the presence of an equilibrium point (called a focus equilibrium) at the center of the limit cycle (green dotted line in Fig. 3B). By applying a varying external (*I*_*app*_) these equilibria can be altered or disappear entirely. If there is the presence of both a stable equilibrium (green solid line Fig. 3B) and a saddle-node equilibrium (blue solid line Fig. 3B), spiking behavior is not possible. Without either of these equilibria present, the cell will oscillate around the focus equilibrium. As the current injection increases, the saddle-node equilibrium and stable equilibrium come closer, until they contact and annihilate. This point, called the saddle-node bifurcation current (*I*_*sn*_, cyan marker Fig. 3B), represents a current threshold necessary for spiking to occur. The saddle equilibrium and stable equilibrium intersect at the saddle-node bifurcation (*I*_*sn*_ = 4.09 pA), which sets the current threshold for spikelet formation. Any current over this limit will cause spiking, while any current under this will cause hyperpolarization. At a resting state (*I*_*app*_ = 0.0pA) the cell is prevented from spiking by the presence of a stable, saddle and a focus equilibrium. Adding sodium currents do not change the spike dynamics but do increase the rate at which the cell depolarizes (Supplemental Fig. 1).

The addition of TREK1 channels adds an extra potassium current. The reversal potential of these channels is that of potassium (*E*_*K*_ =  − 90.0 mV). As the variable *B*_*t*_ increases a negative current will be activated. During a single noisy trial, the average TREK1 current is − 23.3 ± 0.02 pA (Fig. 3C). This pushes the cell into a hyperpolarization that lasts while the variable *B*_*t*_ remains high. The variable *B*_*t*_ increases as the cell is spiking (Fig. 3C). This feedback loop allows for the formation of a saddle equilibrium and prevents further spiking (Fig. 3D).

### Spontaneous channel noise and parameter tuning

Because the saddle-bifurcation is situated above 0pA (*I*_*sn*_ = 4.09 pA), a sufficiently depolarizing event is required for spiking to occur. The initiation of a burst is thought to occur spontaneously^15,17,35^. Noise is simulated by a random walk Ornstein–Uhlenbeck (OU) process (Fig. 3E) and can be sufficiently depolarizing enough to start a burst. This process introduces noisy drift into the equation, meaning that noise has a time dependent component and is partially dependent on noise from the previous time step. The result of this is that noise “events” can accumulate or dissipate. This differs from gaussian noise processes where there is no relationship between noise at two timepoints^36^. This process when added to the neuron creates bursts of events rather than a more tonic spiking. A majority of the noise events are under the current threshold needed to initiate a burst (Fig. 3F grey).

**Figure 4:**
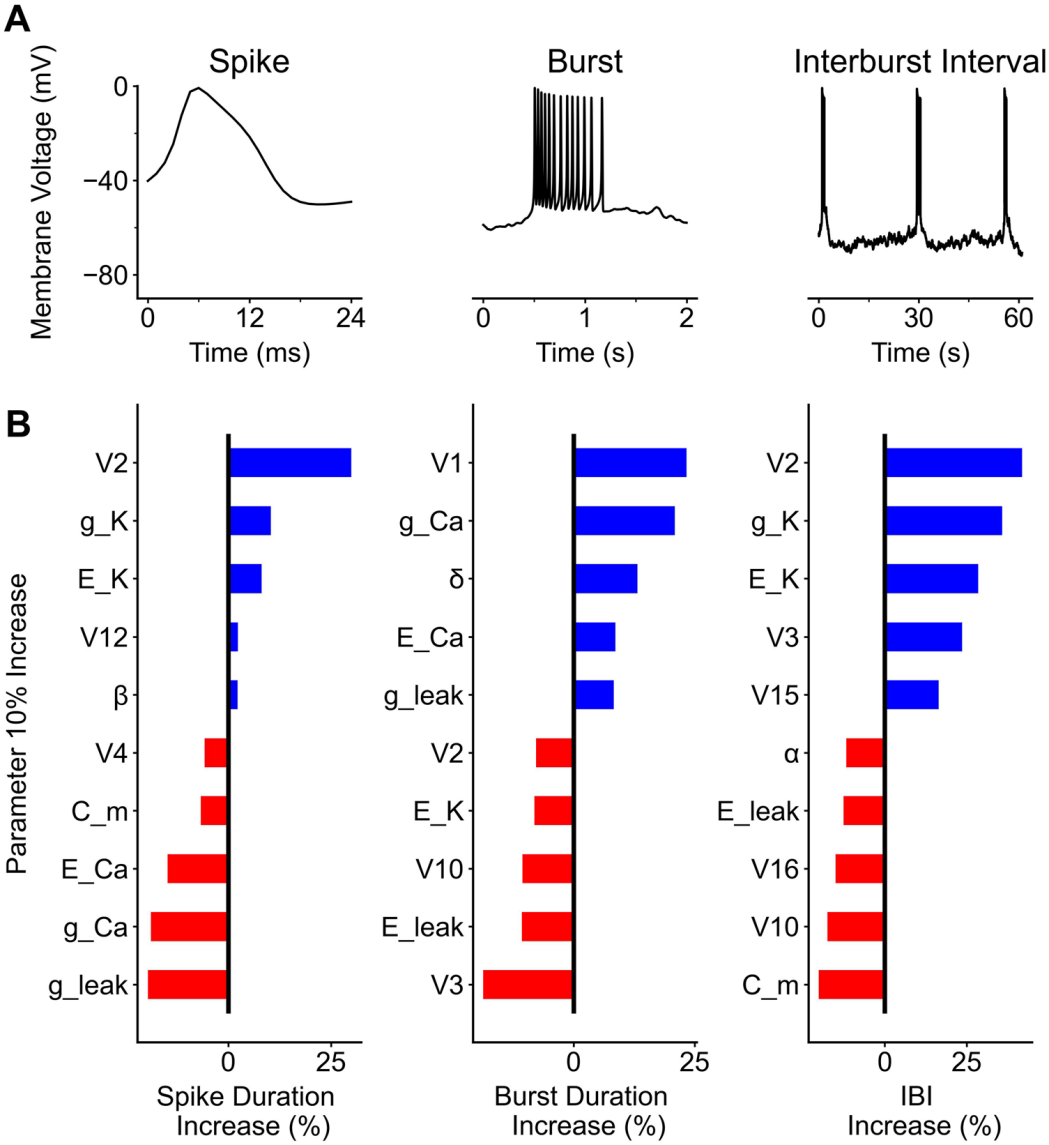
Parameter Gradients and spikes, bursts, and interburst intervals. Examples of a spike, burst, and interburst interval (**A**) are plotted. Each parameter of the model refers to one of 54 different biophysical properties of the model. The value of these parameters was increased by 10%. Parameters that increased the model property are blue, whereas parameters that caused the model property to decrease are in red (**B**). The top 5 increases for each model property are listed, as well as the top 5 decreases listed in descending order.

Noisy models could be used to calculate each of the spike, burst, and IBI parameters. This model includes 54 parameters outlined in Table 2. Three measurements were used to quantify the data: spike duration, burst duration, and the interburst interval. These three measurements represent the three timescales at which physiological events described in the model are occurring. Each of the outlined parameter’s gradient was calculated against each of the three spiking properties of the model (Figure 4A). Model spikes were especially sensitive to the gating parameter of calcium channels (*V*_*2*_), potassium channel conductance and reversal (*g*_*K*_, and *E*_*K*_). Leaky channel conductance (*g*_*Leak*_) reduces the length of the spikes (Figure 4B). Bursts were sensitive to calcium channels properties (*g*_*Ca*_ and *E*_*Ca*_), but also the current dependent rate of calcium influx (*δ*). Because TREK channels are potassium dependent, potassium reversal potential (*E*_*K*_) plays an important role in the length of the interburst interval, while a higher leak potential (*E*_*Leak*_) decreases the time between bursts.

### Neurotransmitter and network dynamics

**Figure 5:**
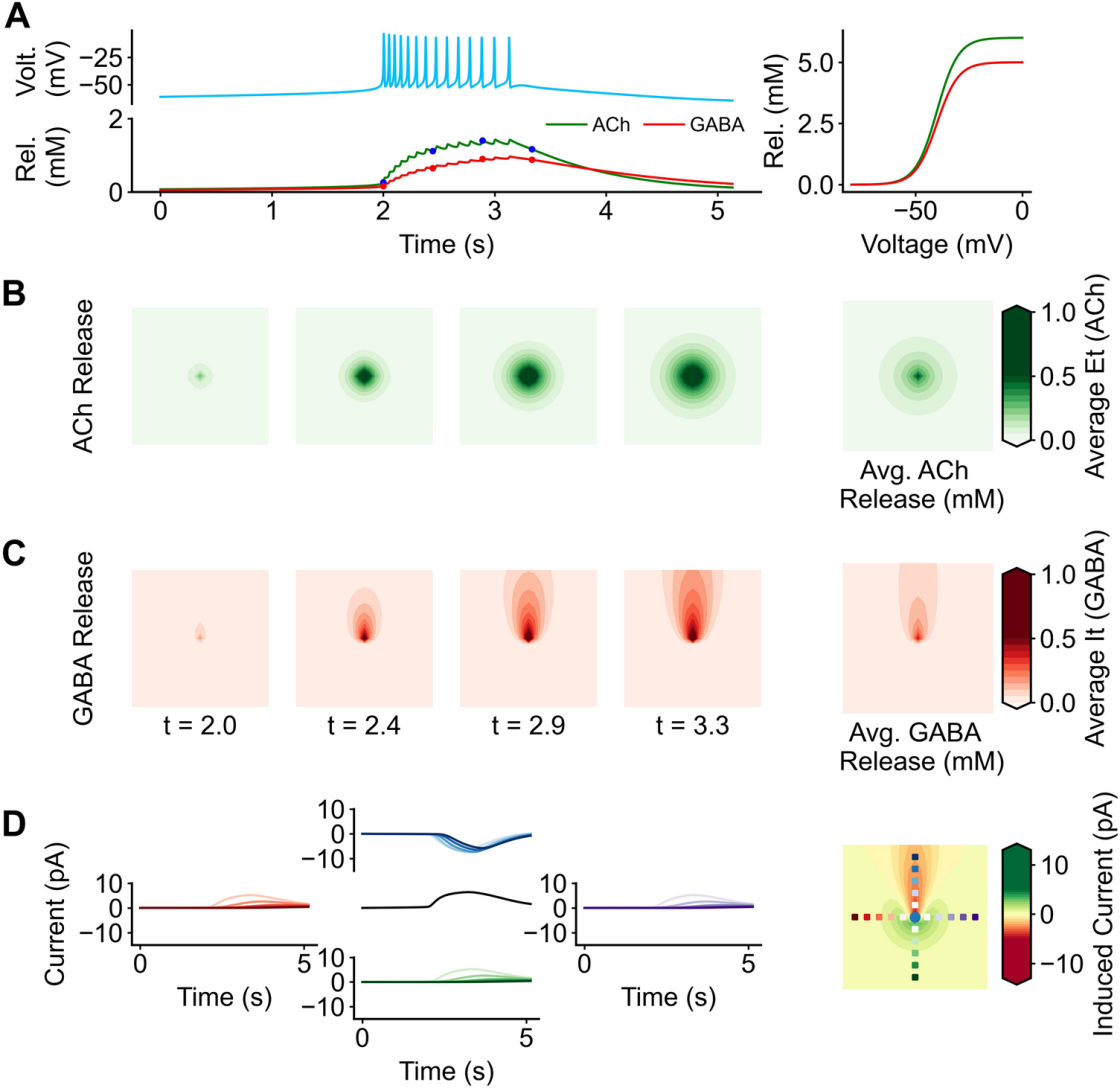
Neurotransmitter dynamics and synchronization. Neurotransmitter release alters the dynamics of the model and allows for cell to synchronize. (**A**) As the voltage (*V*_*t*_) increases over time, acetylcholine (*E*_*t*_) and GABA (*I*_*t*_) are released from the cell according to a sigmoidal function (right panel). This release does not only occur in a temporal manner, but also in a spatial manner. (**B**) Acetylcholine and (**C**) GABA are both released from a central point, but they have different spatial distributions. (B5) and (C5) These values are averaged over time to get a profile of acetylcholine and GABA respectively. (**D**) Cells are sampled at 6 separate sampling spots. Plotted are the currents induced if the receiving cell were to be located at each one of the sampled spots. A spatial profile of induced current by the single cell was made by taking the average currents from the acetylcholine and GABA profiles.

Simulated cells provide input to each other by neurotransmitter release. Previous models have utilized an excitatory neurotransmitter current^20^. Cells that are spiking reach oscillate around the focus equilibrium (Figs. 2B and 3B) at − 58 mV with the upper bounds of the oscillation being − 6 mV. The release of neurotransmitter is modeled using a sigmoidal function that has a half saturation constant at − 40.0 mV. Since this burst is well above that threshold, the cell releases both acetylcholine (*ρe* = 6.0uM/mV) and GABA (*ρi* = 5.0uM/mV) (Fig. 5A). By adding an excitatory current *I*_*ACh*_, and an inhibitory current *I*_*GABA*_ we allow individual cells to interact with each other.

When the ACh receptors are half saturated and the cell is held at V =  − 40.0, the cholinergic current is above the saddle node-threshold (*I*_*ACh*_ = 8.6 pA > *I*_*sn*_ = 4.09) and GABAergic current is below (*I*_*GABA*_ =  − 22.5 pA > *I*_*sn*_ = 4.09). In the presence of acetylcholine, the cell is pushed into a depolarized state, and in the presence of GABA the cell is pushed into a hyperpolarized state. As the sampled cell is further away from the original point of acetylcholine less acetylcholine reaches it. Acetylcholine diffuses outward equally in all directions (Fig. 5B; Supplemental Movie 1A). GABA diffusion is symmetrical on the right and left directions, but some asymmetry is introduced in the top and bottom directions. This results in a majority of GABA forming a tail of diffusion towards the top (Fig. 5C; Supplemental Movie 1B). While the reversal potential for chloride is negative, this results in a hyperpolarization and introduces directionality. Diffusion affects cells closer to the cell releasing the neurotransmitter (Fig. 5D). Cells that are further away from each other have less shared neurotransmitter, and therefore a smaller induced current (whether GABAergic currents, or cholinergic currents) (Supplemental Movie 1C). Cells further away will have a smaller effect on each other. A similar effect occurs for GABA, except cells that are closer will be more inhibited. Altering the conductance for acetylcholine (g_ACh_ = 0.215 µS) and GABA (g_GABA_ = 0.9µS) can alter the shape of this current and will silence or excite the front and back to lesser or greater degrees.

### Comparing physiological data to the model

**Figure 6:**
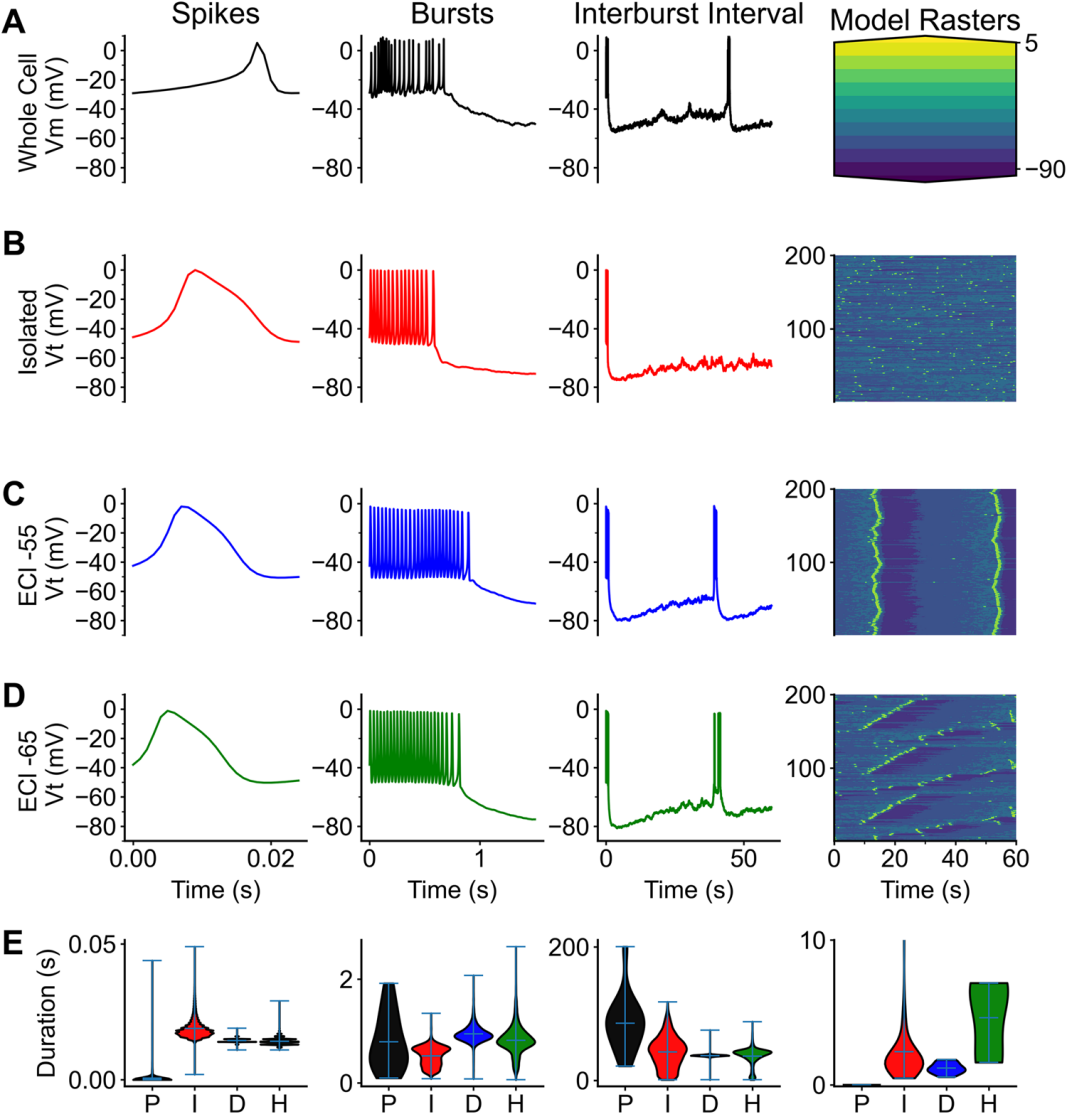
Comparison to physiological data. A comparison of physiological data from three different simulations. Each simulation is compared at 3 different timescales, spikes (0–25 ms), bursts(0–1 s), and interburst interval (0-60 s). (**A**) A representative trace from whole cell patch clamp. (**A**) column 4 is a color scalebar for the raster traced below in column 4. Traces in (**B**) are from a network simulation that does not receive any cholinergic or GABAergic stimulation. Traces in (**C**) result from a network with both GABA and acetylcholine neurotransmitter release. However, the reversal potential for chloride is set to − 55 mV causing GABA to be slightly depolarizing. Traces in (**D**) come from a network with both cholinergic and GABAergic stimulation, but with GABA acting as an inhibitory neurotransmitter. (**E**) Violin plots represent distributions of each dataset using the max interval algorithm. The key for violin plot x labels are: P = physiological, I = no GABA, no acetylcholine, D = depolarizing GABA, H = hyperpolarizing GABA. Column 1 is the spike duration, column 2 is the burst duration, column 3 is interburst interval, column 4 is wave duration.

To understand which behaviors are realistic, the simulated data was compared to physiological data. Each trace was compared to previously collected whole cell patch clamp traces^37^. Raw traces of the data both simulated and physiological were compared. Qualitatively the traces contained the same features as the simulated traces: Spikes sat on top of slowing bursts followed by the sAHP (Fig. 6A).

Four separate simulations were compared (Table 3). One simulation was run without any neurotransmission (Fig. 6B) acetylcholine currents or GABA currents (g_ACh_ = 0.0, g_GABA_ = 0.0). This may compare to a physiological trace where both GABA and Acetylcholine receptors are blocked, or where cells are isolated^18^. This represented cells that were “isolated” and could not synchronize. These simulations lacked any wavelike activity (Supplemental Movie 2). Cholinergic currents were added and compared (Fig. 6C). In these simulations almost all cells participated in a retinal wave as bursts spanned the entire retina (Supplemental Movie 3). Reversal potentials of chloride were set to − 65 mV which makes GABA channels hyperpolarizing and more typical wavelike activity appeared (Fig. 6E; Supplemental Movie 4)^20^. GABAergic currents while reversal potentials were set at − 55 mV shifting GABA currents from hyperpolarizing to excitatory (Fig. 6D; Supplemental Movie 5).

**Table 3:** Comparison of results between an isolated neuron in a network, a lattice neuron simulation, and physiological data collected via whole cell patch clamp.

	No NT (N = 4096)	ACh only (N = 4096)	E_Cl_ = − 55 mV (N = 4096)	E_Cl_ = − 65 mV (N = 4096)	Physiology (N = 5)
Baseline (*mV*)	− 61.08 ± 0.01	− 62.89 ± 0.01	− 63.38 ± 0.01	− 61.15 ± 0.01	− 13.75 ± 6.03
Min Amplitude (mV)	− 77.73 ± 0.05	− 81.63 ± 0.0	− 80.42 ± 0.01	− 80.03 ± 0.02	− 27.9 ± 4.08
Max Amplitude (mV)	− 1.07 ± 0.11	− 0.58 ± 0.0	− 1.66 ± 0.01	− 1.01 ± 0.01	− 0.32 ± 1.48
Spike Duration (ms)	19.18 ± 0.01	13.94 ± 0.0	14.92 ± 0.0	14.22 ± 0.0	14.43 ± 0.31
Burst Duration(ms)	521.89 ± 2.04	729.02 ± 0.83	936 ± 1.50	821.5 ± 2.35	878.04 ± 77.02
IBI(s)	41.91 ± 0.34	38.25 ± 0.01	35.40 ± 21.8	36.42 ± 0.14	65.74 ± 5.87
Wave/Burst Ratio	0.002	0.072	0.016	0.105	N/A
Wave duration (s)	1.16 ± 0.10	5.26 ± 1.07	4.63 ± 1.23	1.7 ± 0.02	N/A
Wave area (cell)	1.2 ± 0.11	2221.71 ± 730.27	2382.2 ± 971.71	12.01 ± 0.95	N/A
Wave velocity (cell/ms)	1.48 ± 0.25	16,164.88 ± 5861.7	15,784.44 ± 6468.47	31.57 ± 3.41	N/A

For modeled data n is equal to the number of simulated cells. For physiological data 1 n is a single whole cell recording. *IBI* Interburst Interval.

Wave activity was compared between 3 simulations. The isolated solution (Fig. 6B4), results in very few events that would be considered a wave (Table 3). The ratio of bursts to waves is 0.002 waves occurring for every 1 burst. Acetylcholine only (data not shown) and the depolarizing (E_Cl_ =  − 55 mV) reversal potential of chloride (Fig. 6C4) are similar in the wave duration, wave area, and wave velocities (Table 3). These simulations tend to generate waves that span all cells. Increasing the equilibrium potential (Fig. 6D4) of chloride (E_Cl_ =  − 65 mV) results in shorter waves, with a smaller wave area. The wave/burst ratio indicates that when GABA plays a hyperpolarizing role, the wave does not cover as much distance.

## Discussion

This model is a synthesis of elements from previous models. The oscillating nature of voltage and potassium repolarization was investigated in the Morris-Lecar model^3^. This model was originally applied to voltage oscillations in the barnacle giant muscle fiber but was applied to fast spiking behavior of starburst amacrine cells^33^. This model was extended by the addition of diffusive behaviors of neurotransmitters including the fractional activation and release of acetylcholine and GABA^20^. Further improvements to this model included the introduction of potassium repolarization variables and a multi-step afterhyperpolarization process^34^. Our model redescribes the second messenger variables in the model as second messenger processes (cAMP/PKA oscillations: *A*_*t*_, *B*_*t*_ respectively). TREK1 potassium channels are likely target for the generation of the sAHP^31,32^. We also introduce a term (I_*t*_) describing the release and activation of inhibitory GABAergic currents*.* This is important for the establishment of directional selectivity in the adult retina and alters the spontaneous activity of SACs^26^. We use an Ornstein–Uhlenbeck process to describe spontaneous burst-like behavior. This noise process is more consistent with neuronal activity^36^. This model introduces sodium currents as well. The addition of voltage gates sodium channels is consistent with previously published data^1,35,38^.

The link between calcium concentration and the sAHP has not been completely described, however TREK1 channels are a possible explanation^29^. This model, as well as being mathematical, places the sAHP generation on the TREK1 activation pathway. This includes a more physiologically plausible pathway to the sAHP. Second messenger systems are present in the SAC cell layer^29,30,39,40^. The most likely pathway for TREK1 activation is the calcium-dependent cAMP decay followed by disinhibition of TREK1 channels. One potential mechanism may be calcium activated phosphodiesterase (PDE) via calmodulin. Similar mechanisms appear in models of the activation of Rods and Cones^41^. This calcium activated decrease in cAMP is the first step in the sAHP, and we include it as *A*_*t*_. PDE inhibition by PDE selective inhibitors MMBX and IBMX altered retinal wave frequencies^19^. Several mechanisms could act to keep cAMP concentration at a steady state. Adenyl cyclase (AC) generates cAMP utilizing adenosine triphosphate (ATP). This mechanism could keep cAMP concentrations at a high steady state^32^. Adenosine may also play a role in the retinal wave frequency and increases calcium transients in the SAC^42^. Forskolin, an adenyl cyclase activator increases the frequency of retinal waves^18,29^. Protein kinase A (PKA) is activated by cAMP at a ratio of 4 cAMP to 2 PKA, and this begins the second part of the sAHP. Both cAMP and PKA oscillate with calcium in SACs^31^. Proteomics and cell culture experiments indicated that TREK1 has two active serine residues capable of phosphorylation. Both sites need to be sequentially phosphorylated for the inactivation of TREK1^30^. Because 4 cAMPs are needed to activate 2 PKAs, and 2 PKAs are needed for complete inactivation of TREK1, this supports the 4th order relationship represented in this model by *B*_*t*_. The connection between Calcium and TREK1 then occurs as the concentration of intracellular calcium increases, cAMP begins to breakdown, PKA concentration decreases, TREK1 can gradually dephosphorylate, and comes out of a nearly steady state of inactivation. This second messenger cascade could lead to potential downstream targets for modulation of the wave frequency.

Directionality of retinal waves later in development could potentially be explained by the presence of GABAergic currents^21,26^. Early presence of cation-chloride channels may result in GABA acting as a hyperpolarizing neurotransmitter and may explain a shift in waves from non-directional to directional^23^. Previous studies have indicated that GABA channels permeable to chloride go through a reversal. Earlier in development in the presence of a cation-chloride exchanger, GABA channels have equilibrium potentials above the resting membrane potential at − 55 mV^23^. Later in development however, the equilibrium potential of chloride has become more hyperpolarizing, and GABA then acts as an inhibitory neurotransmitter. Studies indicate that there may be asymmetric release of GABA which contributes to the direction bias of retinal waves^27,43^. This model agrees with previous publications that predict a lack of directional preference while the equilibrium of chloride channels is depolarizing. GABAergic circuits exist alongside cholinergic circuits and starburst cells are unique in their ability to synthesize both GABA and acetylcholine^22^.

When comparing models, the presence of excitatory and inhibitory neurotransmitter does not affect the spiking properties or bursting properties. One reason could be that the extra excitation occurs on a slow enough time scale that the voltage gated potassium channels could still repolarize the membrane. However, the addition of acetylcholine into the model causes enough excitation to allow the cell to burst more frequently. A decreased IBI could mean the presence of a higher degree of excitatory drive either in the form of cholinergic currents or other mechanisms. While an isolated SAC can only burst for a set amount of time, cells receiving cholinergic stimulation can burst for longer amounts of time. Despite this, some SACs still will display simple bursting behavior. Understanding the physiological sources of the most basic properties of the network could provide insight into the molecular components of this network. Further studies could utilize the ability to fine tune individual parameters and draw comparisons between different experimental models. Further studies could also utilize parameter optimization to tune parameters more directly to data that has been collected from experimentation.

## Methods

### Model formulation

Analysis was conducted for each single cell, regardless of if the cell was isolated or in a lattice. Therefore, lattice simulations consisted of 125 × 96 cells. Each "cell" contains 9 ordinary differential equations, (*V*_*t*_*, N*_*t*_*, M*_*t*_*, H*_*t*_*, C*_*t*_*, A*_*t*_*, B*_*t*_*, E*_*t*_*, I*_*t*_), two a partial differential equation to simulate the 2D diffusion of acetylcholine *∂*^2^*E*_*xy*_ and GABA *∂*^2^*I*_*xy*_, and a stochastic process to represent noisy channel activity *W*_*t*_. Simulated terms consist of the membrane voltage (*V*_*t*_), potassium relaxation (*N*_*t*_), Sodium channel gating (*M*_*t*_), sodium channel inactivation (*H*_*t*_), Calcium concentration (*C*_*t*_), breakdown of cAMP (*A*_*t*_), the TREK1 channel activation (*B*_*t*_), the extracellular concentration of acetylcholine (*E*_*t*_), the extracellular concentration of GABA (*I*_*t*_*)* and a Ornstein–Uhlenbeck process representing noisy channel activity (*W*_*t*_).

The voltage equation is calculated by the summation of all ionic currents *n* = (*Leak, Ca, K, Na, TREK1, ACh, GABA, Noise*) divided by the membrane capacitance. This was calculated from the membrane capacitance from physiological data (*C*_*m*_ = 13.6 *pF*) and applied this to the voltage equation. Each current is calculated with the form *I*_*n*_ =  − *g*_*n*_ ∗ *R* ∗ (*V*_*t*_ − *E*_*n*_), where *g* is the maximal conductance, *R* being the gating fraction and (*V*_*t*_ − *E*_*n*_) being described in the Hodgkin Huxley equations^1^. An applied current (*I*_*app*_) can be used in cases of dynamic investigation and simulates a "current injection" like patch-clamp experiments. to cause the neuron to spike. This can be either something that is applied experimentally, or as a placeholder for unknown currents. Below each current is described as well as the variables that influence it.

1 $${C}_{m}\frac{dV}{dt}={\sum }_{n}{I}_{n}\left({V}_{t}\right)+{I}_{app}$$

$${I}_{leak}\left({V}_{t}\right)=-{g}_{leak}\left({V}_{t}-{E}_{leak}\right)$$

$${I}_{Ca}\left({V}_{t}\right)=-{g}_{Ca}{M}_{\infty }\left({V}_{t},{V}_{1},{V}_{2}\right)\left({V}_{t}-{E}_{Ca}\right)$$

$${I}_{K}\left({V}_{t},{N}_{t}\right)=-{g}_{K}{N}_{t}\left({V}_{t}-{E}_{K}\right)$$

$${I}_{TREK}\left({V}_{t},{B}_{t}\right)=-{g}_{TREK}{B}_{t}\left({V}_{t}-{E}_{K}\right)$$

$${I}_{Na}\left({V}_{t}{M}_{t}{H}_{t}\right)= -{g}_{Na}{{M}_{t}}^{3}{H}_{t}\left({V}_{t}-{E}_{Na}\right)$$

$${I}_{ACh}\left({V}_{t},{E}_{t}\right)=-{g}_{ACh}\overline{H }\left({E}_{t},{\upkappa }_{e}\right)\left({V}_{t}-{E}_{ACh}\right)$$

$${I}_{GABA}\left({V}_{t},{I}_{t}\right)=-{g}_{GABA}\overline{H }\left({I}_{t},{\upkappa }_{i}\right)\left({V}_{t}-{E}_{Cl}\right)$$

$${I}_{noise}\left({W}_{t}\right)=\upsigma {W}_{t}$$

Fractional conductance of the channel is calculated by utilizing a gating factor *R*. For leaky channels, the *R* is a constant 1 which means that all channels are open all the time. For voltage gated channels the fractional proportion of ionic channels for Ca and K are described in the equations for *M*_∞_ and *N*_∞_ respectively.

$${M}_{\infty }\left({V}_{t},{V}_{1},{V}_{2}\right)=\frac{1}{2}tanh\left(\frac{{V}_{t}-{V}_{1}}{{V}_{2}}\right)$$

$${N}_{\infty }\left({V}_{t},{V}_{3},{V}_{4}\right)=\frac{1}{2}tanh\left(\frac{{V}_{t}-{V}_{3}}{{V}_{4}}\right)$$

These equations were derived from the Boltzmann equations relating the internal ionic concentration to the external ionic concentration^1^. *V*_2_ and *V*_4_ represents the midway point where 50% of all ion channels are open, and *V*_1_ and *V*_3_ represents the steepness of the function^3^. Voltage gated potassium channels are independently evolving through repolarization variable, *N*_*t*_.

2 $${\uptau }_{N}\frac{dN}{dt}=\Lambda \left({V}_{t},{V}_{3},{V}_{4}\right)\left({N}_{\infty }\left({V}_{t},{V}_{3},{V}_{4}\right)-N\right)$$

This term is simulated numerically and utilizes the *N*_∞_ gating equation as well as a rate constant:

$$\Lambda \left({V}_{t},{V}_{3},{V}_{4}\right)=cosh\left(\frac{{V}_{t}-{V}_{3}}{2{V}_{4}}\right)$$

Sodium channels are gated by four separate equations. Two corresponding to the gating of sodium channels and two corresponding to the inactivation (*H*_*t*_). Parameters V7-V16 correspond to different properties of the gating equations.

$${\alpha }_{m}\left({V}_{t}\right)= \frac{-({V}_{t}-{V}_{8})}{({V}_{7}\mathrm{exp}(\frac{-{V}_{t}-{\mathrm{V}}_{8}}{{V}_{9}} - 1)}$$

$${\beta }_{m}\left({V}_{t}\right)={V}_{10} \mathrm{exp}\left(\frac{-{V}_{t}-{V}_{11}}{{V}_{12}}\right)$$

3 $$\frac{dM}{dt}={\alpha }_{m}\left({V}_{t}\right)\left(1-{M}_{t}\right)- {\beta }_{m}\left({V}_{t}\right){M}_{t}$$

$${\alpha }_{h}\left({V}_{t}\right)={V}_{13} \mathrm{exp}\left(\frac{-{V}_{t}-{V}_{14}}{{V}_{15}}\right)$$

$${\beta }_{h}\left({V}_{t}\right)= \frac{1}{({V}_{16}\mathrm{exp}(\frac{-{V}_{t}-{\mathrm{V}}_{17}}{{V}_{18}}+ 1)}$$

4 $$\frac{dM}{dt}={\alpha }_{m}\left({V}_{t}\right)\left(1-{M}_{t}\right)- {\beta }_{m}\left({V}_{t}\right){M}_{t}$$

*B*_*t*_ represents the proportion of unphosphorylated TREK1 channels capable of conducting potassium currents and also evolves through two separately evolving numerical values calcium and the Calcium mediated decay of cAMP *C*_*t*_ and *A*_*t*_:

5 $${\uptau }_{C}\frac{dC}{dt}={C}_{0}+\updelta {I}_{Ca}\left({V}_{t}\right)-\uplambda {C}_{t}$$

Calcium concentration is dependent on the Calcium currents calculated above in the voltage equation. As the cell continues to spike, calcium influx increases at a rate dependent on the voltage (δ = 0.010503 mM/mV). To prevent the equation from reaching negative concentrations of calcium *C*_0_ represents a minimal calcium concentration^34^. Intracellular buffers such as [Cl-] and Calmodulin cause a decrease in calcium represented by *λC*_*t*_. Overtime calcium concentration will decrease, and this is represented by *τ*_*C*_. We describe *A*_*t*_ as the calcium mediated breakdown of cAMP.

6 $${\uptau }_{A}\frac{dA}{dt}=\mathrm{\alpha }{{C}_{t}}^{4}\left(1-{A}_{t}\right)-{A}_{t}$$

This model describes a potential mechanism for Calcium dependent breakdown of cAMP requires 4 Calcium molecules^32^. For this reason, *C*_*t*_ is taken to the 4th power. The breakdown of cAMP prevents the gradual phosphorylation of TREK1 to pTREK1, and so *A*_*t*_ influences *B*_*t*_ which acts as the sAHP gating value. The value for *A*_*t*_ increases dependent on calcium (*C*_*t*_) at a rate of *α* = 625. *A*_*t*_ decays at a rate of *τ*_*A*_ = 8300* ms.* Once *A*_*t*_ reaches a threshold, *B*_*t*_ begins to increase as well at a rate of *β* = 34 and decays with a time constant of *τ*_*B*_ = 10000* ms*.

7 $${\uptau }_{B}\frac{dB}{dt}=\upbeta {{A}_{t}}^{4}\left(1-{B}_{t}\right)-{B}_{t}$$

Once a voltage threshold is reached, the cell will begin to release acetylcholine and GABA according to the equation

8 $${\uptau }_{E}\frac{dE}{dt}={D}_{e}{{\nabla }_{\{dXe, dYi\}}}^{2}{E}_{t}+\mathrm{\rho e\Phi }\left({V}_{t},{V}_{se},{V}_{0e}\right)-{E}_{t}$$

9 $${\uptau }_{I}\frac{dI}{dt}={D}_{i}{{\nabla }_{\{dXi, dYi\}}}^{2}{I}_{t}+\mathrm{\rho i\Phi }\left({V}_{t},{V}_{si},{V}_{0i}\right)-{I}_{t}$$

Acetylcholine and GABA are both released according to the sigmoidal function Φ

$$\Phi \left({V}_{t},{V}_{s},{V}_{0}\right)=\frac{1}{1+exp\left[-{V}_{s}\left({V}_{t}-{V}_{0}\right)\right]}$$

Over a 2D grid, acetylcholine (*E*_*t*_) and GABA (*I*_*t*_) spread outwards according to a Crank–Nicholson diffusion stencils *De*∇^2^ and *Di*∇^2^. The rate of diffusion in both acetylcholine (*D*_*e*_ = 0.005 mM/ms) and GABA (*D*_*i*_ = 0.005 mM/ms) describes how quickly the neurotransmitters will move outward. A lower number means a slower diffusion. These variables can be adjusted to allow for faster simulated flow. When this is multiplied by the vector for *E*_*t*_ and *I*_*t*_, this simulates transmission of either ACh or GABA over X and Y coordinates. Acetylcholine has no differences in the diffusion variables in the left to right direction (*dXe* = {1.0, 1.0}) or top to bottom (*dYe* = {1.0, 1.0}). GABA spreading is not biased in the right and left (*dXi* = {1.0, 1.0}), but is biased in the top to bottom directions (*dYi* = {1.9, 0.1}).

The activity caused by acetylcholine and GABA are described by a similar function: the fractional activation of the receptors channels in the presence of neurotransmitters *E*_*t*_ or *I*_t_. To describe the diffusion of neurotransmitters we utilized a partial differential equation that describes the spatial diffusion of neurotransmitters.

$$\overline{H }\left({E}_{t},{\kappa }_{e}\right)=\frac{{E}_{t}^{2}}{{E}_{t}^{2}+{{\kappa }_{e}}^{2}}$$

Parameters dXe, dYe, dXi, and dYi are vectors that represent the directionality bias of diffusion. By setting both to 1.0 in the case of acetylcholine, this means the acetylcholine propagates outward on a sphere. This agrees with previous studies, where retinal waves have a directional bias to retinal waves toward the nasal side^27^. In this case the parameter dYe = {0.1, 1.9}, which represents 90% of the acetylcholine activity will be in the downward (or nasal) direction relative to the lattice.

This noise process is controlled by an amplitude gain (*σ* = 0.1pA), and noise dissipation (*τ*_*w*_ = 800.0 ms).

10 $${\uptau }_{W}\frac{dW}{dt}=-{W}_{t}$$

### Code compilation and running

The code was compiled and run in the high-level numeric computing language Julia^44^. The package DifferentialEquations.jl was used to build all the ordinary differential equations. Simulations were run using a stability optimized stochastic integration algorithm (SOSRI)^45^. The models were simulated for 300 s (5 min) using adaptive time-stepping methods. During a dense simulation (throughout which every timestep was saved), trajectories generated 244,000 data points per 300,000 timesteps and had an average timestep of 1.2 ms (equivalent to 1.2 kHz). For both isolated single trace analysis and lattice simulations, the saving interval was set to 1 ms, (equivalent to 1 kHz acquisition). The simulations were run in a 64-bit Windows 10 Operating system with Intel(R) Core (TM) i7-1065G7 CPU @ 2.80 GHz with 16.00 GB of RAM. Single trace simulations took 20 s-40 s to run. 3D simulations with a grid of 64 × 64 cells took on average 10 min to run. The length of time each simulation is run varies slightly based on the frequency of spikes occurring.

### Electrophysiology

Whole-cell patch clamp experiments were performed as previously described^37^. Voltage traces from I = 0 were digitized with a sampling rate of 20 kHz.

### Timescale analysis

Timescale analysis was conducted on patch-clamp traces, and the voltage values of the simulation (*V*_*t*_). For the whole-cell patch-clamp traces, a validation trace was printed with bursts labeled. Only traces that contained both a burst and a sAHP were chosen to include in the analysis. Each cell in the lattice simulation was considered an individual trace and was analyzed independently.

The minimum and maximum and baseline amplitude of each trace was recorded. The threshold was found by taking the average voltage value of the trace and then adding 4 × the standard deviation. Next the trace was converted into a true/false vector, with values above the threshold being true, and values below the threshold being converted into false. A grouping function was then applied to consecutive true values, which were then converted into spike timestamps. The average spike duration was calculated by returning the time between the start timestamp and the end timestamp. After this, a max interval algorithm as described in^13,17^ was applied using these parameters. This algorithm grouped spikes together into groups or "bursts" based on fixed spiking parameters. The parameters for the max interval spike sorting are listed in (Table 4). The burst timestamps of each cell in the simulation were obtained from the max interval algorithm. These timestamps were used to calculate the burst duration. The distance between the end of one burst and the beginning of the next was used to calculate the interburst interval (IBI). The spike durations, burst durations, and interburst intervals for each simulation were compiled into a list, and then the mean and standard deviation on these lists were calculated. Lists were populated with a single cell in a single cell simulation, and all cells in a lattice simulation. All analysis was done in custom compiled code written in Julia language. All functions can be found in the module wave_analysis.jl.

**Table 4:** Max interval spike sorting properties used by the burst calculation algorithm.

Spike property	Value	Units
*ISIstart*	500	ms
*ISI*_*end*_	500	ms
*IBImin*	1000	ms
*Duration*_*min*_	100	ms
*SPB*_*min*_	4	spikes

*ISI* Interspike Interval, *IBI* Interburst Interval, *SPB* spiked per burst.

### 2D wave analysis

After the max interval algorithm was applied, bursting patterns were then analyzed. Simulations were converted to 3D array with cells x, y, and time coordinate. Image segmentation was used on the 3D array. This links all bursts that are consecutive either in the x, y, or time dimension. A list was compiled for each wave/burst event that included all the x, y, and time coordinates included in that event. If the event was limited to one x and y coordinates, then it was excluded from the wave calculations. The elapsed time of each wave was calculated from the minimum time coordinate vs the maximum time coordinate. The area travelled by the wave was calculated by the total x distance travelled by the total y distance. Finally, the wave velocity was calculated by taking the area travelled multiplied by the velocity. Units were in cells/s.

### Parameter gradients on spike, burst, and interburst interval properties

Each parameter of the model refers to one of 54 different biophysical properties of the model (values and units for each parameter are outlined in Table 2). To determine the gradients, the spike, burst, and interburst interval were calculated from 40 repeated simulations. Parameters were then increased by 10% and 40 successive trials were run. The model properties were averaged over each of these 40 trials. The gradient was then calculated by taking the percent of the initial parameter value to the final parameter value and then subtracted by 100%. Results are indicated in Table 5. Increases resulted in positive percentage increases, while decreases resulted in negative percent. These were calculated for spike durations, burst durations, and interburst intervals.

**Table 5:** Parameter gradients versus each of the model properties.

Par	Spike (%)	Burst (%)	IBI (%)
*V*_1_	− 0.26	23.3	7.78
*V*_2_	29.9	− 7.77	41.9
*V*_3_	− 2.22	− 18.66	23.57
*V*_4_	− 5.78	− 0.83	1.85
*V*_*0e*_	0.14	− 3.17	1.17
*V*_*0i*_	− 0.02	− 6.32	− 7.50
*V*_*se*_	0.51	3.90	7.51
*V*_*si*_	1.23	2.97	15.35
*V*_*7*_	2.21	− 1.89	− 2.87
*V*_*8*_	0.20	1.40	9.29
*V*_*9*_	− 2.47	2.98	0.77
*V*_*10*_	− 0.20	− 10.56	− 17.48
*V*_*11*_	− 3.79	− 5.48	− 3.20
*V*_*12*_	2.31	− 2.58	5.55
*V*_*13*_	0.24	− 1.26	1.43
*V*_*14*_	0.71	− 0.60	12.16
*V*_*15*_	0.76	7.70	16.41
*V*_*16*_	− 0.32	1.62	− 15.03
*V*_*17*_	− 0.76	0.37	− 7.69
*V*_*18*_	0.49	7.07	12.79
*C*_*m*_	− 6.70	− 6.64	− 20.15
*g*_*Leak*_	− 19.61	8.32	− 3.52
*g*_*Ca*_	− 18.82	20.92	− 5.43
*g*_*K*_	10.33	5.24	35.84
*g*_*Na*_	− 1.40	2.02	4.84
*g*_*TREK*_	1.46	− 1.37	8.91
*g*_*ACh*_	− 0.53	− 5.22	− 11.91
*g*_*GABA*_	3.25	4.05	16.59
*E*_*Leak*_	− 3.57	− 10.69	− 12.60
*E*_*Ca*_	− 14.82	8.65	− 4.95
*E*_*K*_	8.11	− 8.07	28.51
*E*_*Na*_	− 0.01	− 0.91	− 0.79
*E*_*ACh*_	1.38	− 6.04	− 2.55
*E*_*Cl*_	1.16	− 3.02	− 2.38
*τn*	− 4.45	− 0.47	5.37
*τa*	− 2.03	− 1.07	6.13
*τb*	− 1.59	− 2.41	− 11.53
*τc*	− 0.68	0.49	5.02
*τACh*	0.36	0.00	1.26
*τGABA*	0.93	2.72	7.04
*τW*	1.21	1.83	3.58
*C*_0_	0.72	− 2.95	− 4.12
*λ*	− 0.89	− 3.11	− 5.95
*δ*	− 3.66	13.18	− 5.08
*α*	0.24	− 5.23	− 11.78
*β*	2.25	− 0.49	5.38

Gradients are indicated as percent changes.

## Supplementary Information

Supplementary Figure 1.

Supplementary Figure 2.

Supplementary Legends.

Supplementary Video 1.

Supplementary Video 2.

Supplementary Video 3.

Supplementary Video 4.

Supplementary Video 5.

## Data Availability

The scripts needed to run all the simulations are available at: https://github.com/mattar13/RetinalChaos.
