# Supplementary figures and images for: Modeling cholinergic retinal waves: starburst amacrine cells shape wave generation, propagation, and direction bias
Source: Sci Rep. 2023 Feb 17;13:2834. doi: 10.1038/s41598-023-29572-2 (PMC9938278; doi:10.1038/s41598-023-29572-2)

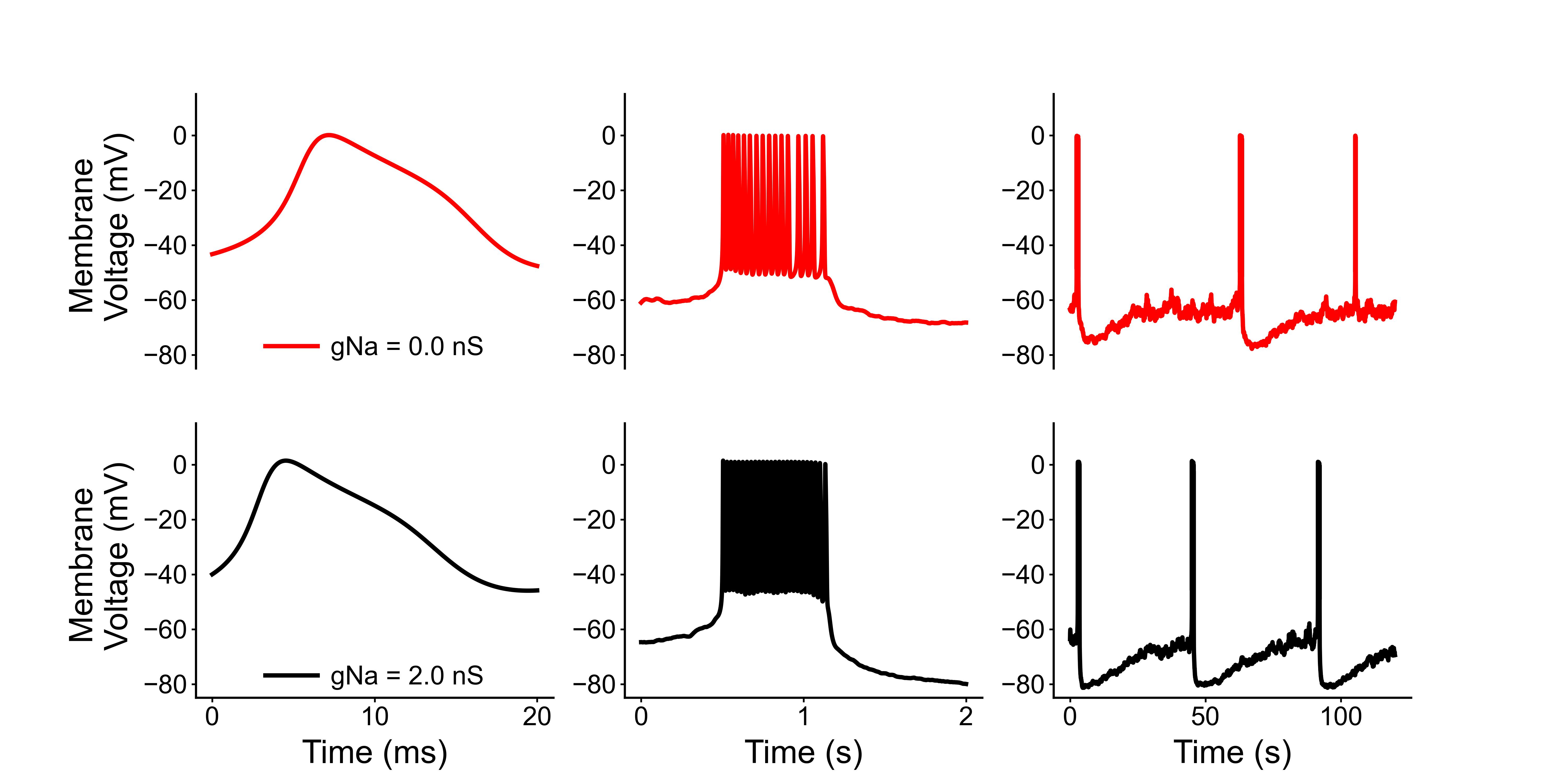

Supplement: Supplementary file 1 — Supplementary Figure 1. [file 41598_2023_29572_MOESM1_ESM.jpg]

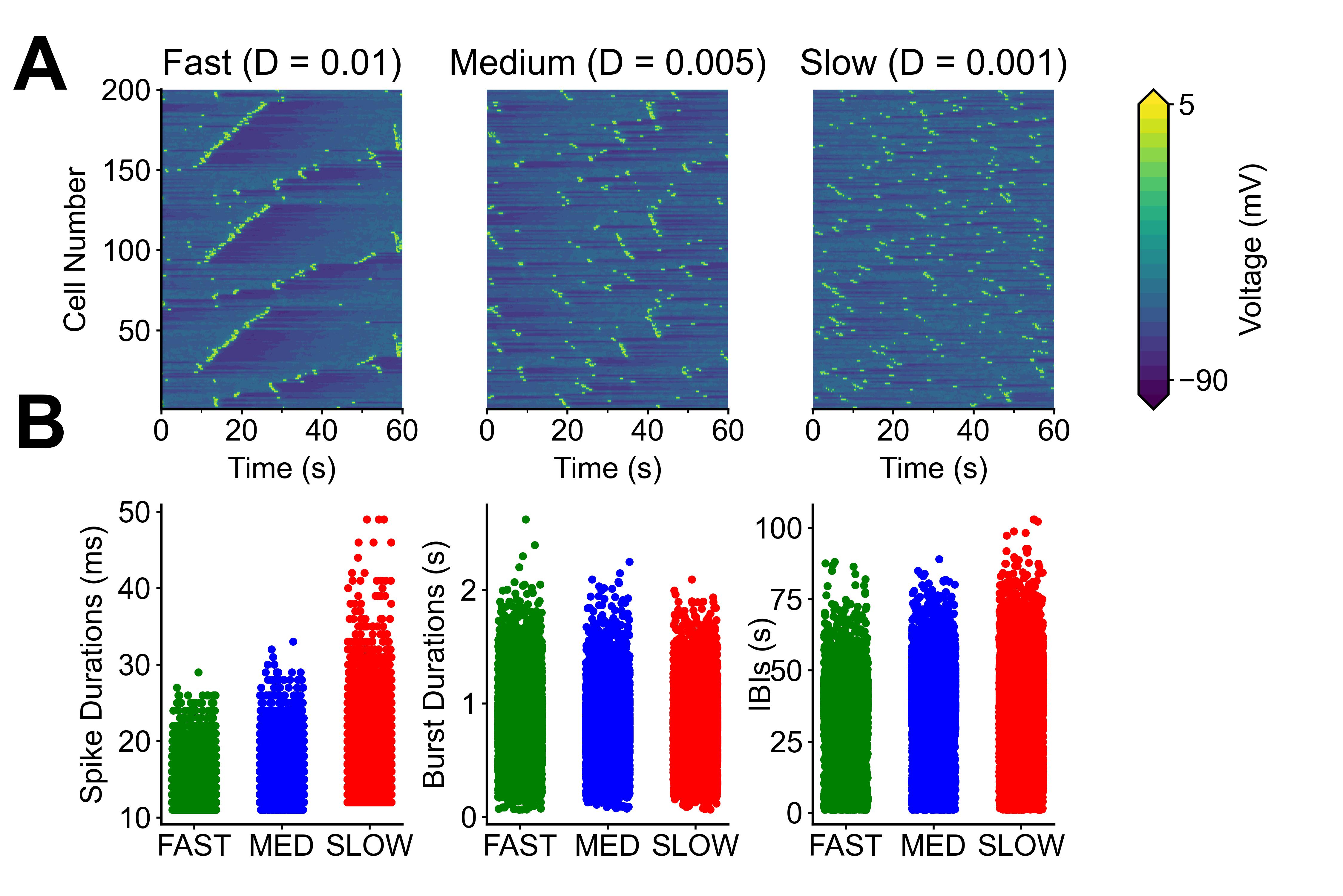

Supplement: Supplementary file 2 — Supplementary Figure 2. [file 41598_2023_29572_MOESM2_ESM.jpg]
