# Supplementary material for: Modeling cholinergic retinal waves: starburst amacrine cells shape wave generation, propagation, and direction bias
Source: Sci Rep. 2023 Feb 17;13:2834. doi: 10.1038/s41598-023-29572-2 (PMC9938278; doi:10.1038/s41598-023-29572-2)
Supplement: Supplementary file 3 — Supplementary Legends. [file 41598_2023_29572_MOESM3_ESM.docx]

**Supplementary Figure 1 Voltage gated sodium channel dynamics**

Two simulations were run. The red traces have removed the influence of the voltage gated sodium channels (*g_Na_* = 0.0 nS). The black traces on the other hand have included a small sodium channel conductance (*g_Na_* = 2.0nS). The biophysical properties of these channels are similar.

**Supplementary Figure 2 Speeds of synaptic spread.**

The speed of synaptic spread was altered. Synaptic spreading ranged from fast (*D_e_* and *D_i_*  = 0.01mM/ms) to medium (*D_e_* and *D_i_*  = 0.005mM/ms) to slow (*D_e_* and *D_i_*  = 0.001mM/ms). A raster of each simulation was shown in (**A**) with the color of each pixel. Model properties (spike duration, burst duration, and interburst interval) were compared between diffusion speeds (**B**).

**Supplementary Movie 1 Neurotransmitters and induced currents**

An example of the diffusion of GABA and Acetylcholine. A single cell model is run with the default parameters with an injected current (*I_app_*) of 15 pA. (**A**)The outward diffusion of Acetylcholine occurs equally in all directions. (**B**) GABA diffusion contains a “tail” representing a diffusion bias in the top direction. This can be altered to go in any direction. (**C**) The current represents the current induced by neurotransmitter release from an activated SAC was calculated. This calculation was done using a voltage value of -40mV as the holding potential. That value was chosen because it is past the half release constant for both acetylcholine (*V_0e_*) and GABA (V*_­0i_*).

**Supplementary Movie 2 All neurotransmission blocked**

A simulation was run without the influence of neurotransmitters. This was done by setting the parameters for acetylcholine receptor conductance and GABA receptor conductance to 0. (*g_ACh_ = 0.0, g_GABA_ = 0.0*). Simulation sizes were 64x64 cells large. Simulations were run for 60s (60,000 ms) as a warmup, and then simulated for 120s.

**Supplementary Movie 3 No GABA transmission blocked**

A simulation was run without the influence of the inhibitory neurotransmitter GABA. This was done by setting the GABA receptor conductance to 0 (*g_GABA_ = 0.0*). Simulation sizes were 64x64 cells large. Simulations were run for 120s (120,000 ms) as a warmup, and then simulated for 120s.

**Supplementary Movie 4 Hyperpolarizing GABA channel simulation**

A simulation was run of the default parameters with the equilibrium potential of chloride set to -65mV. Simulation sizes were 64x64 cells. Simulations were run for 60s (60,000 ms) as a warmup, and then simulated for 120s.

**Supplementary Movie 5 Depolarizing GABA channel simulation**

A simulation was run after changing the equilibrium potential of chloride to -55 mV. Simulation sizes were 64x64 cells. Simulations were run for 60s (60,000 ms) as a warmup, and then simulated for 120s.
